# Matrine alleviates depressive-like behaviors via modulating microbiota–gut–brain axis in CUMS-induced mice

**DOI:** 10.1186/s12967-023-03993-z

**Published:** 2023-02-24

**Authors:** Ming Zhang, Aoqiang Li, Qifang Yang, Jingyi Li, Lihua Zheng, Guannan Wang, Ying Sun, Yanxin Huang, Muqing Zhang, Zhenbo Song, Lei Liu

**Affiliations:** 1 National Engineering Laboratory for Druggable Gene and Protein Screening, Northeast Normal University, Changchun, China; 2 Hubei Key Laboratory of Genetic Regulation and Integrative Biology, School of Life Sciences, Central China Normal University, Wuhan, China; 3 School of Molecular & Cellular Biology, University of Illinois Urbana Champaign, Urbana, IL USA

**Keywords:** Matrine, Depression, Gut microbiota, Metabolomics, Microbiota–gut–brain axis

## Abstract

**Background:**

The realization of the “microbiota–gut–brain” axis plays a critical role in neuropsychiatric disorders, particularly depression, is advancing rapidly. Matrine is a natural bioactive compound, which has been found to possess potential antidepressant effect. However, the underlying mechanisms of regulation of the “microbiota–gut–brain” axis in the treatment of depression by oral matrine remain elusive.

**Methods:**

Its antidepressant effects were initially evaluated by behavioral tests and relative levels of monoamine neurotransmitters, and matrine has been observed to attenuate the depression-like behavior and increase neurotransmitter content in CUMS-induced mice. Subsequently, studies from the “gut” to “brain” were conducted, including detection of the composition of gut microbiota by 16S rRNA sequencing; the metabolomics detection of gut metabolites and the analysis of differential metabolic pathways; the assessment of relative levels of diamine oxidase, lipopolysaccharide, pro-inflammatory cytokines, and brain-derived neurotrophic factor (BDNF) by ELISA kits or immunofluorescence.

**Results:**

Matrine could regulate the disturbance of gut microbiota and metabolites, restore intestinal permeability, and reduce intestinal inflammation, thereby reducing the levels of pro-inflammatory cytokines in peripheral blood circulation and brain regions, and ultimately increase the levels of BDNF in brain.

**Conclusion:**

Matrine may ameliorate CUMS-induced depression in mice by modulating the “microbiota–gut–brain” axis.

**Supplementary Information:**

The online version contains supplementary material available at 10.1186/s12967-023-03993-z.

## Background

Depression is a chronic recurrent emotional disorder and physiological disease that has a high global incidence. The World Health Organization (WHO) identified depression as the third leading cause of the global burden of disease in 2008, and predicts that depression will become the first global burden of disease by 2030 [1]. Although depression has an obvious biological basis, its pathogenesis is complicated and not fully understood [2]. Therefore, it is crucial to develop the additional pathological mechanisms of depression.

Following the publication of Jane A. Foster’s article on how the microbiome influences anxiety and depression through the gut–brain axis in 2013 [3], the interaction between the gut microbiota and the brain has gradually become the focus of neuroscience research. Successive studies have reported that the gut microbiota of patients with depression is different from that of healthy people [4], and depression-like behaviors lead to changes in the diversity and abundance of gut microbiota [4, 5], which suggests that depression affects the composition of the gut microbiota. In addition, transplanting the fecal microbiota of patients with major depression into sterile mice exhibited depression symptoms [6], and the recovery of the gut microbiota alleviates depression [7], indicating that changes in the gut microbiota may promote the onset of depression and play a vital role in the pathophysiology of depression. It is worth noting that metabolic disorders have recently been recognized as a characteristic of depression, in which the gut microbiota leads to changes in the intestinal metabolome [8], thereby alleviating intestinal metabolic disorders has become a potential target of antidepressant. The gut microbiota intimately connects the gut and the brain, and regulates the physiology and cognitive function of the brain through neural, endocrine, or immune pathways [9, 10]. Especially in CUMS-induced mice, the inflammatory immune response is closely involved in the bidirectional communication between the gut and the brain [11]. However, the pathogenesis of depression is complicated that the drugs used clinically still have the problem of single therapeutic direction and unsatisfactory therapeutic effects.

Matrine is a quinolizidine alkaloid derived from the traditional Chinese medicine *S. alopecuroides*. Studies indicate that matrine has anti-inflammatory and neuroprotective effects [12, 13], and exerts antidepressant-like effects on CUMS-induced depression mice by promoting hippocampal PI3K/Akt/mTOR signaling [14]. In addition, our previous studies have shown that the total alkaloids of *S. alopecuroides* with matrine as one of the main components can improve the depression in CUMS-induced mice via modulating the gut microbiota [15]. Accordingly, the purpose of present study is to investigate whether the improvement effect of matrine on the depression-like behavior of CUMS-induced model mice involves gut microbiota through 16S rRNA sequencing, metabolomic detection, and multiple biological detection techniques, and to explore its probable pathways in the “microbiota–gut–brain” axis.

## Materials and methods

### Animals

The experimentations were performed in male ICR mice weighing (18–22 g) obtained from Liaoning Changsheng Biotechnology Co., Ltd. (Changchun, China). The mice were housed in the state of controlled environmental conditions (12 h light/dark cycle; 21 °C ± 1 °C temperature; 52% ± 2% relative humidity), and given a standard diet and water ad libitum.

### Chronic unpredictable mild stress and drug treatments

CUMS procedure was performed as described previously with a slight modification [15]. After one week of adaptation, the mice were housed in a separate cage and exposed four weeks of CUMS procedure. The detailed CUMS stressors are as follows: food deprivation for 12 h; water deprivation for 12 h; lights on at night for 12 h; empty cage for 12 h; wet bedding for 12 h; confinement in a tube for 2 h; traffic noise (70–90 dB) for 6 h; cage tilting for 12 h (45°); exposure to a stroboscope for 12 h; foreign body stimulation for 6 h; crowding for 12 h (ten mice within one cage); level shaking for 15 min; food and water deprivation for 24 h (Additional file 1: Table S1). In brief, two different types of mild stressor are applied to mice every day, which varied from day to day to make the stress procedure unpredictable.

**Fig. 1:**
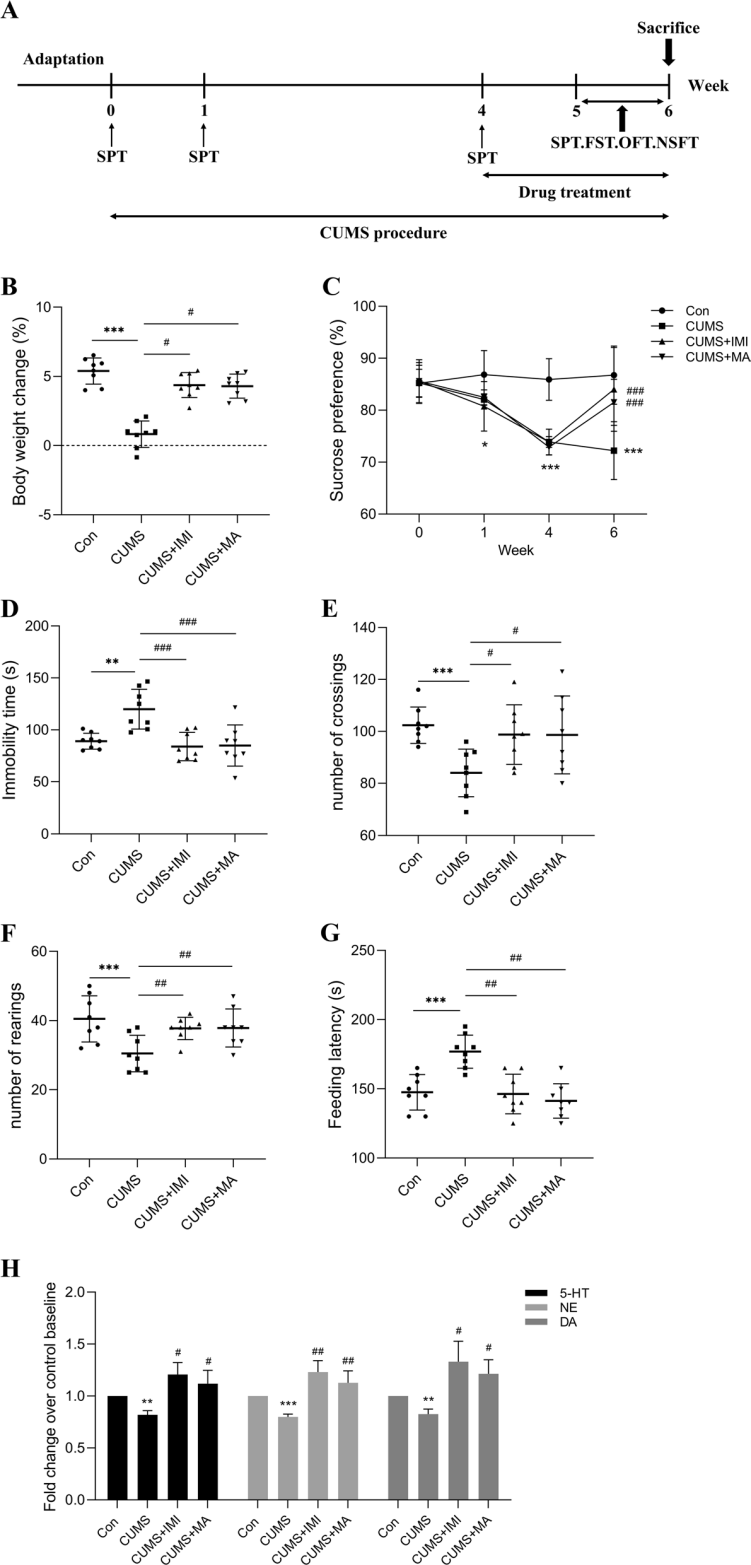
Body weight change, depressive-like behaviors, and monoamine neurotransmitter levels in different groups. **A** The schematic representation of experiment schedule. **B** Body weight change (%) in week 6. **C** Sucrose preference (%) in the SPT. **D** Immobility time in the FST. **E** The number of crossings in the OFT. **F** The number of rearings in the OFT. **G** The feeding latency in the NSFT. **H** The relative levels of monoamine neurotransmitter (5-HT, NE, and DA) in serum. Data are presented as the mean ± SEM (n = 8). *p_*adj*_ < 0.05, **p_*adj*_ < 0.01, ***p_*adj*_ < 0.001 versus the control group (Con); ^#^*p*_*adj*_ < 0.05, ^##^*p*_*adj*_ < 0.01, ^###^*p*_*adj*_ < 0.001 versus the CUMS group

Mice were randomly divided into six groups with 10 in each group: Control group (Con, 0.9% physiological saline), CUMS model group (CUMS, 0.9% physiological saline), CUMS + imipramine (CUMS + IMI, 30 mg/kg), CUMS + Low/Medium/High-dose matrine (CUMS + 15 mg/kg; 30 mg/kg; 60 mg/kg MA). Matrine and imipramine were purchased from Yuanye Biological Co., Ltd. (Shanghai, China) and prepared in 0.9% physiological saline. Imipramine was used as a positive control in this study. Except for the intraperitoneal injection of the mice in the imipramine group, the mice in other groups were given intragastric administration. After the CUMS mouse depression model was successfully established, matrine and imipramine were administered every morning at a fixed time (9:00–10:00), and the CUMS procedure was performed 30 min after the drugs were given. The specific experimental time schedule of this study is shown in Fig. 1A.

### Body weight and behavioral test

#### Body weight

The body weight (BW) of mice was recorded every day, and the weight at week 0 was used as the baseline value. The change in BW in week 6 was calculated as follows: the change in BW/baseline BW × 100%.

#### Sucrose preference test

The sucrose preference test (SPT) was conducted according to previously described [15]. As shown in Fig. 1A, the SPT was performed at four time points: the adaptation period (week 0), before the drug treatment (weeks 1 and 4) and before the animals were sacrificed (week 6). First, train the mice for 48 h to adapt to the 1% sucrose (Chemical Industry Group Co., Ltd., Beijing, China) solution (w/v) and avoid the preference for bottle placement: two bottles of 1% sucrose water were given each cage for 24 h adaptation, replaced one bottle of 1% sucrose water with pure water for 12 h, and then exchange the positions of the two bottles for 12 h. Next, the mice were deprived of food and water for 24 h. Then SPT was performed: two new bottles were randomly placed, filled with 1% sucrose water and pure water, respectively. The mice drank freely for 2 h, and the water bottles were weighed before and after placing them. Sucrose preference rate was calculated as follows: the intake of sucrose solution/the total amount of liquid consumed × 100%.

#### Forced swimming test

The forced swimming test (FST) was carried out as previously described [15]. Briefly, each mouse was individually placed in a cylindrical plastic container (25 cm height, 10 cm diameter) filled with 20 cm deep of 25 ± 1 °C water in a quiet environment and forced to swim for 6 min. Record and count the immobility time after 4 min by digital camera. After the test, each mouse was carefully dried and the equipment was rinsed with water. The time in which the mice gave up struggling to float on the water surface or only slightly swing their limbs to prevent immersion in the water was counted as immobility time.

#### Open field test

The open field test (OFT) was performed in accordance with previous procedure [15]. Briefly, the mice were placed individually in the center of black open box (30 cm × 30 cm × 30 cm) in a quiet environment. The box consisted of sixteen equal squares on the bottom. The experimental light source was from indoor lighting. Each mouse was free to move for 6 min. The activity of the mouse in the last 5 min were videotaped for further parameter analysis, including recording the number of crossings and rearings.

#### Novelty-suppressed feeding test

The novelty-suppressed feeding test (NSFT) was carried out as previously described with slight modification [16]. Briefly, the test was performed for 2 consecutive days: the first day is the adaptation period and animals were individually placed in a black open box for 10 min. After 24 h of food deprivation, three food pills were placed in the center of the open box on the second day, and then the animal was placed in a corner of the box (keeping the same position and direction each time), and test for 5 min. The latency to feed (the time from the start of the test to the first bite of the food) was observed and record. The equipment was cleaned with 10% ethanol after each test.

### Sample preparation

After the last administration, the mice were sacrificed by decapitation. The blood was collected with a vacuum tube (containing ethylenediaminetetraacetic acid) and centrifuged (4 °C, 3000 rpm, 15 min), and then the serum was stored at − 80 °C until assay. Their whole brains were quickly removed and placed in ice-cold physiological saline, the hippocampus and prefrontal cortex tissue were rapidly dissected on the ice surface and immediately frozen in liquid nitrogen until assay. The intestinal contents of the cecum were rapidly collected and immediately stored by liquid nitrogen for later DNA and metabolites extraction.

### Enzyme-linked immunosorbent assay (ELISA) analysis

ELISA kits (Enzyme-linked Biotechnology Co., Ltd., Shanghai, China)were employed to measure the concentrations of serotonin (5-HT), norepinephrine (NE), dopamine (DA), diamine oxidase (DAO), lipopolysaccharide (LPS), interleukin-1β (IL-1β), interleukin-6 (IL-6), tumor necrosis factor-α (TNF-α), and brain-derived neurotrophic factor (BDNF). The experimental operation was carried out according to the manufacturer’s instructions, and the detection was performed using a microplate reader (EnVision, PerkinElmer, USA) at a wavelength of 450 nm.

### Immunofluorescence staining

The mice were deeply anesthetized by intraperitoneal injection of 4% chloral hydrate (Chemical Industry Group Co., Ltd., Beijing, China), and then perfused with 4% paraformaldehyde (PFA, Chemical Industry Group Co., Ltd., Beijing, China). The whole brain tissue was taken out and fixed in 4% PFA at room temperature and protected from light for 12 h, and then dehydrated in 30% sucrose at 4 °C for 48 h. The brain was sliced in the coronal plane (5 μm thick) and subjected to immunofluorescence processing according to the routine procedures in our laboratory. The primary antibody BDNF (1:400, Proteintech, USA) and Cy3-labeled fluorescent secondary antibody (1:1000; Proteintech, USA) was used. The slides were observed under a confocal microscope (LSM880, Zeiss, Germany). The image was analyzed by Image J software for optical density.

### DNA extraction and 16S rRNA gene sequence analysis

DNA was extracted from each intestinal content sample (n = 8 per group) using DNeasy PowerWater Kit (QIAGEN, Inc., Netherlands) following manufacturer’s protocol. The quantity and quality of extracted DNAs were measured using a NanoDrop ND-1000 spectrophotometer (Thermo Fisher Scientific, Waltham, USA) and agarose gel electrophoresis, respectively. Then the V3–V4 hypervariable region of the 16S rRNA gene was PCR amplified using universal primers 338F and 806R [15]. The PCR amplicons were purified, quantified, and sequenced on the Illumina NovaSeq-PE250 platform at Shanghai Personal Biotechnology Co., Ltd., China.

16S rRNA sequences were analyzed using QIIME2 [17]. The DADA2 plugin [18] in QIIME2 was used to quality filtering, dereplicating and chimera filtering, as well as for generation of amplicon sequence variant (ASV) table and representative sequences. Then the representative sequences were assigned based on the Greengenes database 13.8 [19]. A tree was next generated, using the align-to-tree-mafft-fasttree command, and Alpha diversity were produced by QIIME2 core-metrics-phylogenetic pipeline.

### Metabolite extraction and UPLC-Q-TOF-MS analysis

The extraction and analysis of intestinal contents metabolites (n = 8 per group) was assisted by the Beijing Allwegene Technology Co., Ltd. (Beijing, China). The metabolomics separation was performed on a Waters ACQUITY UPLC BEH Amide column (2.1 × 100 mm, 1.7 μm, Milford, Massachusetts, USA). The mobile phase consisted of 25 mM ammonium acetate and 25 mM ammonium hydroxide in water (A) and acetonitrile (B). The following mobile phase gradient is: 95% B, 0.5 min; 95–65% B, 0.5–7 min; 65–40% B, 7–8 min; 40% B, 9 min; 40–95% B, 9–9.1 min; and 95% B, 12 min. The flow rate was 0.5 mL/min. Injection volume was 2 µL and the column temperature was maintained at 30 °C. Mass spectrometry was carried out on the UPLC-Q-TOF-MS (Triple TOF 5600+, SCIEX, USA) through an electrospray ionization (ESI) source operated in positive and negative modes. The MS conditions were set as follows: ion spray voltage: ± 5500 V; ion gas temperature: 650 °C; ion gas pressure: 60 psi; curtain gas: 30 psi; declustered voltage: 60 V. The measurement procedures, acquisition and processing of UPLC-Q-TOF-MS are described in previous studies [20, 21].

### Statistical analysis

16S rRNA gene sequence data analysis: the Kruskal–Wallis test was carried out to compare the differences in alpha diversity between pairs of groups, including Chao 1, Faith’s Phylogenetic diversity, and Shannon, followed by Dunn’s multiple comparisons test with correction via Bonferroni. Then beta diversity was calculated based on Bray-Curtis dissimilarity and visualized using principal coordinate analysis (PCoA) and unweighted pair group method with arithmetic mean (UPGMA). Permutational multivariate analysis of variance (PERMANOVA) with 999 permutations was preformed to assess differences in beta diversity using *adonis()* function in vegan package [22]. The *P* values for multiple comparisons were corrected using Bonferroni correction. The taxonomic changes at the phylum and genus levels between different groups were visualized as column plots by Metastats [23]. Further, linear discriminant Analysis (LDA) effect size (LEfSe) was implemented to identify different ASVs among different treatment groups.

Metabolomics data analysis: Standardized metabolomics data were fed to R package metaX for principal component analysis (PCA) and orthogonal partial least squares discriminant analysis (OPLS-DA). The metabolites with VIP (Variable Importance in the Projection) > 1 and *p* < 0.05 (student t test) were considered as significantly changed metabolites. In addition, MetaboAnalyst 3.0 (http://www.metaboanalyst.ca/) was used to conduct pathway enrichment analysis for the resulting significant differential metabolites and KEGG (http://www.kegg.jp) was used to identify related pathways of differential metabolites.

Spearman correlation analysis was used to explore the correlations between depression-related indicators and gut-related indicators, as well as brain-related indicators and gut-related indicators in mice.

Statistical analysis of the remaining data: All data were expressed as the mean ± standard error of the mean (SEM). One-way analysis of variance (ANOVA) was used for data processing. The *p* values for multiple comparisons were corrected using Bonferroni correction.

## Results

### Matrine treatment alleviated the depression-like behavior and promoted the release of monoamine neurotransmitter in CUMS-induced mice

After four weeks of CUMS induction, mice were given different concentrations of matrine by gavage for 2 weeks. Body weight, behavioral tests (SPT, FST, OFT, and NSFT), and the levels of monoamine neurotransmitters were next conducted to examine the antidepressant effect of matrine. As illustrated in Additional file 1: Fig. S1, after 4 weeks of CUMS induction, the sucrose preference rate of mice was significantly reduced in the 4th week (*p*_*adj*_ = 9.52e−04), indicating that the CUMS modeling was successful. Meanwhile, 30 mg/kg (*p*_*adj*_ = 8.94e−04) and 60 mg/kg (*p*_*adj*_ = 0.03) instead of 15 mg/kg matrine significantly increased the reduction of sucrose preference in the 6th week (ANOVA: SPT, F = 17.36, df = 5, *p* = 5.01e−06). Combined with the results of previous studies [14], 30 mg/kg was finally selected as the optimal dose of matrine for subsequent experiments.

As shown in Fig. 1B–G, body weight gain, anhedonia, behavioral despair, and exploratory ability of mice induced by CUMS for 6 weeks undergone major changes, which are reflected in the reduction of body weight gain (*p*_*adj*_ = 8.78e−04) and sucrose preference rate (*p*_*adj*_ = 6.71e−04), the prolonged FST immobility time (*p*_*adj*_ = 0.009), the reduction of OFT crossings (*p*_*adj*_ = 7.91e−04) and rearings (*p*_*adj*_ = 9.81e−04) number, and the prolongation of NSFT latency (*p*_*adj*_ = 8.33e−04). However, after treatment with imipramine or matrine, the aforementioned depression-like symptoms were improved with statistical significance (ANOVA: BW, F = 9.16, df = 3, *p* = 0.0002; FST, F = 9.32, df = 3, *p* = 0.0002; OFT crossings, F = 8.63, df = 3, *p* = 0.0006; OFT rearings, F = 10.42, df = 3, *p* = 9.02e−05; NFST, F = 12.53, df = 3, *p* = 8.25e−05). It suggests that two weeks of matrine treatment could alleviate depressive-like behaviors of mice.

Depression induced by stress is not only manifested in depression-like behaviors, but also closely related to low levels of monoamine neurotransmitters (5-HT, NE, and DA). The relative content of monoamine neurotransmitters has been used as an important indicator for evaluating depression [24]. Thus, the levels of monoamine neurotransmitters (5-HT, NE, and DA) in the serum were detected. The results are shown in Fig. 1H, CUMS exposure significantly reduced (*p*_*adj*_ = 0.006, *p*_*adj*_ = 9.17e−04, and *p*_*adj*_ = 0.007) the levels of monoamine neurotransmitters in the serum of mice, while treatment with imipramine (*p*_*adj*_ = 0.04, *p*_*adj*_ = 0.006, and *p*_*adj*_ = 0.03) or matrine (*p*_*adj*_ = 0.03, *p*_*adj*_ = 0.008, and *p*_*adj*_ = 0.02) almost reversed the decreasing trend (ANOVA: 5-HT, F = 5.203, df = 3, *p* = 0.004; NE, F = 10.29, df = 3, *p* = 9.14e−05; DA, F = 5.12, df = 3, *p* = 0.005) suggesting that matrine restored the CUMS-induced the decrease of monoamine neurotransmitters levels, which was beneficial to the improvement of depression in mice.

### Matrine modulated the gut microbiota composition in depression-like mice induced by CUMS

**Fig. 2:**
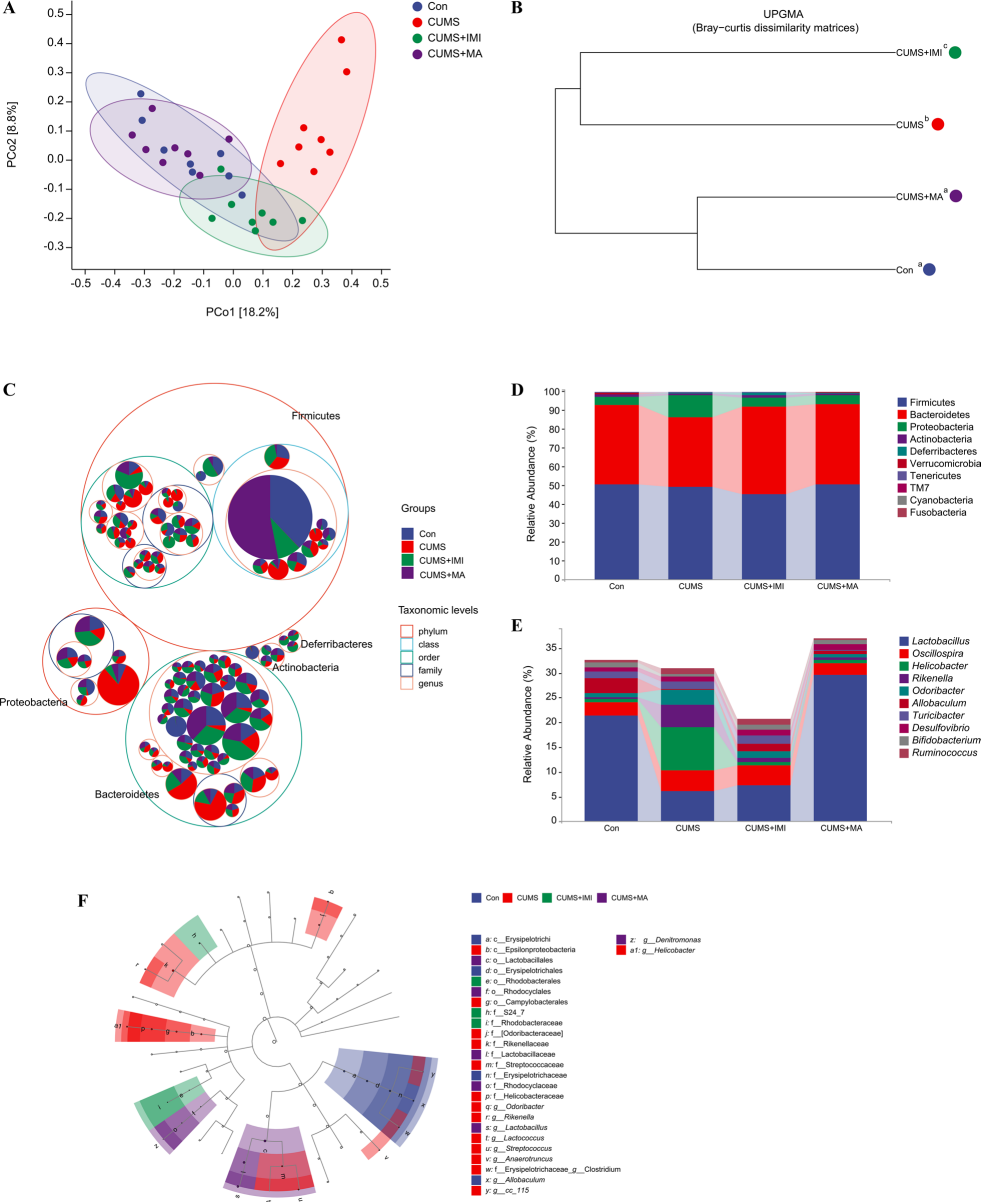
The diversity and composition of gut microbiota in different groups. **A** Principal coordinate analysis (PCoA) plot of beta diversity in different groups by Bray-Curtis distance. Percentages of variance explained by first two axes explain were provided, accounting for 18.2% and 8.8%, respectively. **B** Unweighted pair group-method with arithmetic means (UPGMA) based on Bray-Curtis dissimilarity matrices. Groups with different letters are significantly different according to PERMANOVA (*p*_*adj*_ < 0.05). **C** Taxonomic tree in packed circles. The circles represent phylum, class, order, family, and genus from outside to inside. The abundance of each ASV in the different treatment groups is displayed as a pie chart. **D** Relative abundances of the top ten ASVs at the phylum level. **E** Relative abundances of the top ten ASVs at the genus level. **F** Cladogram showed ASVs with LDA scores greater than 2 based on linear discriminant analysis effect size analysis

To explore the effect of matrine, which can improve depression, on gut microbiota, 16S rRNA sequencing analysis was performed on the microbes in the cecal contents of mice. We compared the alpha diversity and found that there was no significant difference between each pair of groups (Additional file 1: Fig. S2). Further analysis of beta diversity, PCoA revealed that samples were largely separated by groups, which means that the gut microbiota in different treatment groups were significantly different, except for between matrine group and control group (PERMANOVA: Con-CUMS: F = 3.47, *p*_*adj*_ = 0.012; CUMS-CUMS + IMI: F = 2.74, *p*_*adj*_ = 0.006; CUMS-CUMS + MA: F = 4.31, *p*_*adj*_ = 0.006; Con-CUMS + MA: F = 1.62, *p*_*adj*_ = 0.144; Fig. 2A). Consistently, UPGMA clustering further indicated differences between CUMS and CUMS + IMI groups branching away from Con and CUMS + MA groups, while Con and CUMS + MA groups clustered together (Fig. 2B). The above results indicated that the effect of matrine on the gut microbiota of CUMS-induced mice was closer to that of the control group.

The mean relative abundance profiles of gut microbiota composition in each group as overview is shown in Fig. 2C. Firmicutes, Bacteroidetes, and Proteobacteria were the most abundant phyla in all groups, accounting for more than 90% of the total bacterial community (Fig. 2D). At genus level, compared with the control group, the relative abundances of *Lactobacillus* and *Allobaculum* were significantly decreased in the CUMS group, accompanied by a significantly increased in the relative abundances of *Rikenella* and *Odoribacter* (all *p*_*adj*_ = 0.02; Fig. 2E). Compared with the CUMS group, the relative abundance of *Lactobacillus* (*p*_*adj*_ = 8.16e−04) in the matrine treatment group was increased, while the relative abundance of *Helicobacter*, *Rikenella*, *Odoribacter*, and *Ruminococcus* was significantly decreased (all *p*_*adj*_ = 0.03). Notably, among the microbiota with significant changes at the level of the top 10 genera in relative abundance, *Lactobacillus*, *Allobaculum*, *Turicibacter*, *Bifidobacterium*, and *Ruminococcus* are gram-positive bacteria, while *Oscillospira*, *Helicobacter*, *Rikenella*, *Odoribacter*, and *Desulfovibrio* are gram-negative bacteria. The CUMS procedure reduced the total relative abundance of Gram-positive bacteria by more than 50%, while the relative abundance of all Gram-negative bacteria was up-regulated. However, this situation was reversed by matrine treatment, indicating that matrine treatment modulated the gut microbiota composition in CUMS-induced depression mice.

Based on LEfSe, ASVs driving differences in gut microbiota composition between different treatment groups were analyzed. A total of 27 differences ASVs among the four groups were identified (LDA > 2.0), of which 11 were at the genus level (Fig. 2F). The results showed that the different ASVs at the genus level were concentrated in the CUMS group. In detail, the relative abundances of *Odoribacter*, *Rikenella*, *Lactococcus*, *Streptococcus*, *Anaerotruncus*, *Clostridium*, *CC-115*, and *Helicobacter* in CUMS group were significantly different from those in other groups. *Lactobacillus* and *Denitromonas* were the most abundant in matrine group. The dominant ASV in the control group was *Allobaculum*. After analysis, it was found that the differential ASVs in LEfSe (top 50 at the genus level) are *Odoribacter*, *Rikenella*, *Lactobacillus*, *Streptococcus*, *Anaerotruncus*, *Clostridium*, *Allobaculum*, *Denitromonas*, *Helicobacter*. The changes in the composition and abundance of the above-mentioned mice gut microbiota among the different groups are especially shown in Additional file 1: Fig. S3.

### Matrine attenuates gut metabolite disturbances and metabolic pathway changes in CUMS-induced mice

**Fig. 3:**
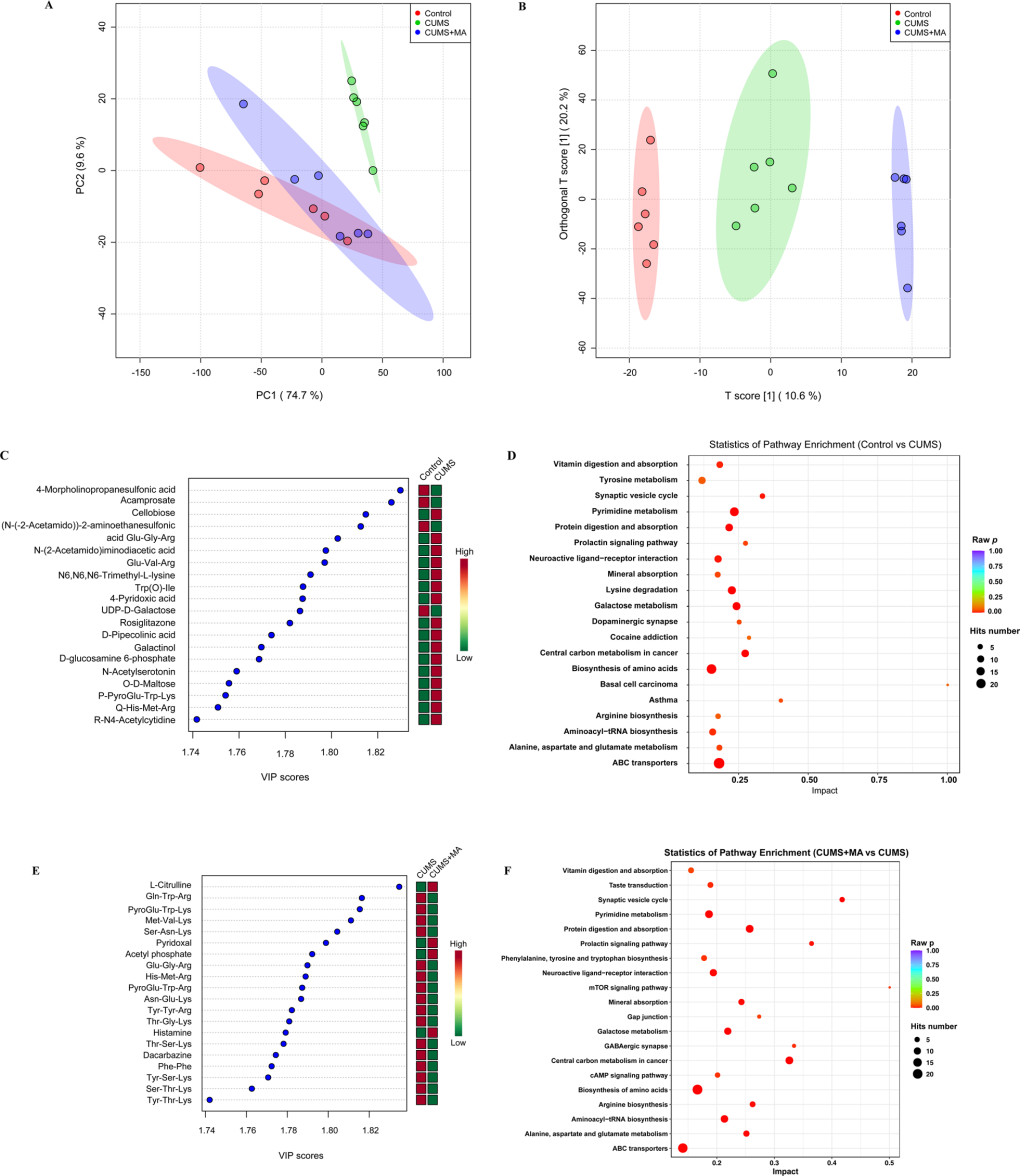
The composition and difference of gut metabolites in different groups, and the analysis of metabolic pathways. **A**, **B** The PCA and OPLS-DA analysis of control group, CUMS model group and matrine treatment group. **C**, **E** The VIP value plots of the differential metabolites (the top 20 of VIP value). **D**, **F** The plot of KEGG differential metabolic pathway (the top 20 hits number of metabolic pathways)

Alters in the composition of gut microbes will lead to changes in gut metabolites [8], then the effect of matrine on gut metabolites was further explored. First, principal component analysis (PCA) and orthogonal partial least squares discriminant analysis (OPLS-DA) were used to analyze the metabolites of the control group, the CUMS group and the matrine group, and the results demonstrated that there were clear differences between the three groups (Fig. 3A, B).

To find the differential metabolites between the groups, a combination of VIP > 1 and *p* < 0.05 was screened in the OPLS-DA. After comparing the control group and the CUMS group, the top 20 differential metabolites with VIP values were selected for display (Fig. 3C). Compared with the control group, the contents of 4 differential metabolites in the CUMS group were significantly increased, while the contents of 16 differential metabolites decreased significantly. Similarly, the differential metabolites in the top 20 VIP values between the CUMS model group and the matrine-treated group were shown in Fig. 3E. Compared with the model group, the contents of 16 differential metabolites increased significantly after matrine treatment, and the contents of 4 metabolites including histamine decreased significantly. Notably, histamine is closely related to the occurrence of inflammation, and the enriched metabolic pathway is inflammatory mediator regulation of TRP channels [25]. Excitingly, in Additional file 1: Table S2, we found that after matrine treatment, the change trend of differential metabolites except Glu-Val-Arg, Trp(O)-Ile, UDP-d-galactose, PyroGlu-Trp-Arg, Thr-Gly-Lys, Phe-Phe, and Ser-Thr-Lys can be reversed. It reflects that matrine reversed most of the changes in gut metabolites in CUMS-induced depression mice.

In addition to the top 20 differential metabolites in the VIP value, we found 7 differential metabolites related to amino acid metabolism: l-phenylalanine, l-tyrosine, l-tryptophan, serotonin (5-HT), norepinephrine (NE), dopamine (DA), and l-glutamine. l-Phenylalanine can be converted into l-tyrosine, and then into NE and DA [26]. l-Tryptophan is the synthetic precursor of 5-HT, and its relative content in the intestine is negatively correlated with intestinal inflammation [27, 28]. In addition to reducing intestinal permeability, l-glutamine can also reduce inflammation and form a strong intestinal barrier [29]. As shown in Additional file 1: Fig. S4, compared with the control group, the levels of the 7 metabolites in the model group showed a decreasing trend. This trend of amino acid disorders was reversed after matrine treatment, confirming the positive regulation of matrine on monoamine neurotransmitters, and implying the possibility of intestinal permeability destruction and inflammation.

Furthermore, to understand the underlying molecular functions of the gut differential metabolites, metabolic pathway analysis was conducted. Figure 3D, F show the differential metabolic pathways (top 20 hits) between the control group and the CUMS group, and the CUMS group and the matrine group, respectively. Among them, there are 14 identical metabolic pathways, namely: ABC transporters; biosynthesis of amino acids; pyrimidine metabolism; neuroactive ligand-receptor interaction; aminoacyl-tRNA biosynthesis; protein digestion and absorption; galactose metabolism; vitamin digestion and absorption; central carbon metabolism in cancer; mineral absorption; alanine, aspartate and glutamate metabolism; arginine biosynthesis; synaptic vesicle cycle; and prolactin signaling pathway. It is suggested that matrine restores most of the abnormal metabolic pathways caused by CUMS.

To explore the correlation between depression-related indicators and gut-related indicators, Spearman correlation analysis was performed on gut metabolites, gut microbiota, and depression-like behaviors (Additional file 1: Fig. S5). We found significant correlations between the above indicators, suggesting that the ameliorating effect of matrine on depressive-like behaviors in CUMS-induced mice involves modulation and restoration of gut microbiota and metabolites. However, the probable related mechanisms of matrine affecting the gut microbiota and metabolites in the treatment of depression need to be further confirmed.

### Matrine reduced the intestinal permeability marker and lowered the levels of LPS in depression-like mice induced by CUMS

**Fig. 4:**
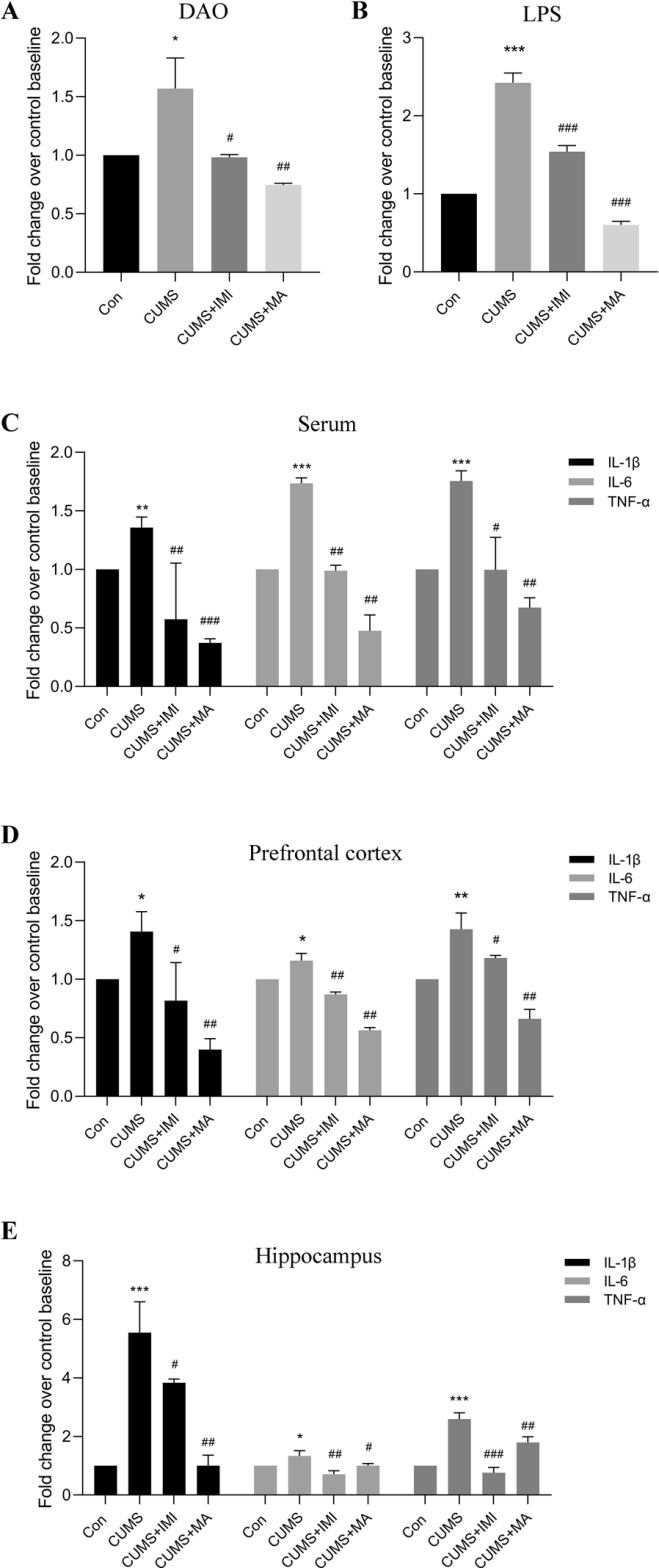
The levels of DAO, LPS, and pro-inflammatory cytokines in different groups. **A**–**C** The relative levels of DAO, LPS, and pro-inflammatory cytokines in serum. **D**, **E** The relative levels of pro-inflammatory cytokines in prefrontal cortex and hippocampus. Data are presented as the mean ± SEM (n = 8). *p_*adj*_ < 0.05, **p_*adj*_ < 0.01, ***p_*adj*_ < 0.001 versus the control group (Con); ^#^*p*_*adj*_ < 0.05, ^##^*p*_*adj*_ < 0.01, ^###^*p*_*adj*_ < 0.001 versus the CUMS group

Since CUMS induced the increase of Gram-negative bacteria (such as *Odoribacter*, *Rikenella*, *Helicobacter*, etc.) in the gut of mice, as well as altered amino acids and their metabolites (such as l-tryptophan, histamine, l-glutamine, etc.), which are closely related to intestinal barrier damage and intestinal inflammation [28–36]. Subsequent ELISA results of DAO, a marker of intestinal permeability in serum, confirmed that the relative content of DAO (Fig. 4A, *p*_*adj*_ = 0.04) in the CUMS group was significantly higher than that in the control group, suggesting that the intestinal permeability of CUMS-induced mice is increased. Since LPS is an important component of gram-negative bacteria, the relative level of LPS was tested. The results of LPS in serum showed that LPS in the CUMS group was significantly increased compared with the control group (Fig. 4B, *p*_*adj *_= 5.68e−04), indicating that mice in the CUMS group may have intestinal inflammation and then release LPS into the blood. However, matrine treatment reversed the increase in DAO (*p*_*adj*_ = 0.03 and *p*_*adj*_ = 0.006) and LPS (*p*_*adj*_ = 7.42e−04 and *p*_*adj*_ = 5.13e−04) level (ANOVA: DAO, F = 5.76, df = 3, *p* = 0.003; LPS, F = 19.87, df = 3, *p* = 3.25e−06), indicating that matrine has anti-inflammatory effects, which in turn restores intestinal permeability and the relative level of LPS.

### Matrine lowered the levels of pro-inflammatory cytokines in peripheral blood circulation and specific brain regions in depression-like mice induced by CUMS

Next, we further tested the levels of pro-inflammatory cytokines in serum and specific brain regions (prefrontal cortex and hippocampus). In the serum, as displayed in Fig. 4C, CUMS procedure significantly increased the relative levels of IL-1β (*p*_*adj*_ = 0.008), IL-6 (*p*_*adj*_ = 5.42e−04), and TNF-α (*p*_*adj*_ =6.35e−04) compared with the control group. Matrine reversed the increase in IL-1β (*p*_*adj*_ = 8.83e−04), IL-6 (*p*_*adj*_ = 0.007), and TNF-α (*p*_*adj*_ = 0.006) compared with the CUMS model group (ANOVA: IL-1β, F = 11.02, df = 3, *p* = 8.51e−05; IL-6, F = 8.94, df = 3, *p* = 0.0003; TNF-α, F = 8.86, df = 3, *p* = 0.0005). Additionally, the relative content of pro-inflammatory cytokines was also measured in the prefrontal cortex and hippocampus of mice, and the trend was similar to that in the serum. As shown in Fig. 4D, E, the relative levels of IL-1β, IL-6, and TNF-α in the prefrontal cortex (*p*_*adj*_ = 0.03, *p*_*adj*_ = 0.02, and *p*_*adj*_ = 0.005) and hippocampus (*p*_*adj*_ = 7.29e−04, *p*_*adj*_ = 0.02, and *p*_*adj*_ = 4.37e−04) of the CUMS group were significantly higher than those in the control group. Matrine decreased the levels of pro-inflammatory cytokines in the prefrontal cortex (*p*_*adj*_ = 0.003, *p*_*adj*_ = 0.002, and *p*_*adj*_ = 0.007) and hippocampus (*p*_*adj*_ = 0.003, *p*_*adj*_ = 0.04, and *p*_*adj*_ = 0.008) (ANOVA: Prefrontal cortex: IL-1β, F = 5.24, df = 3, *p* = 0.004; IL-6, F = 6.20, df = 3, *p* = 0.002; TNF-α, F = 7.14, df = 3, *p* = 0.001; Hippocampus: IL-1β, F = 8.19, df = 3, *p* = 0.0003; IL-6, F = 5.29, df = 3, *p* = 0.004; TNF-α, chi-squared = 9.41, df = 3, *p* = 0.0002), indicating that matrine alleviates the inflammation of peripheral blood circulation and specific brain regions caused by CUMS.

### Matrine reversed the reduction of BDNF levels in the specific brain regions of CUMS-induced mice

**Fig. 5:**
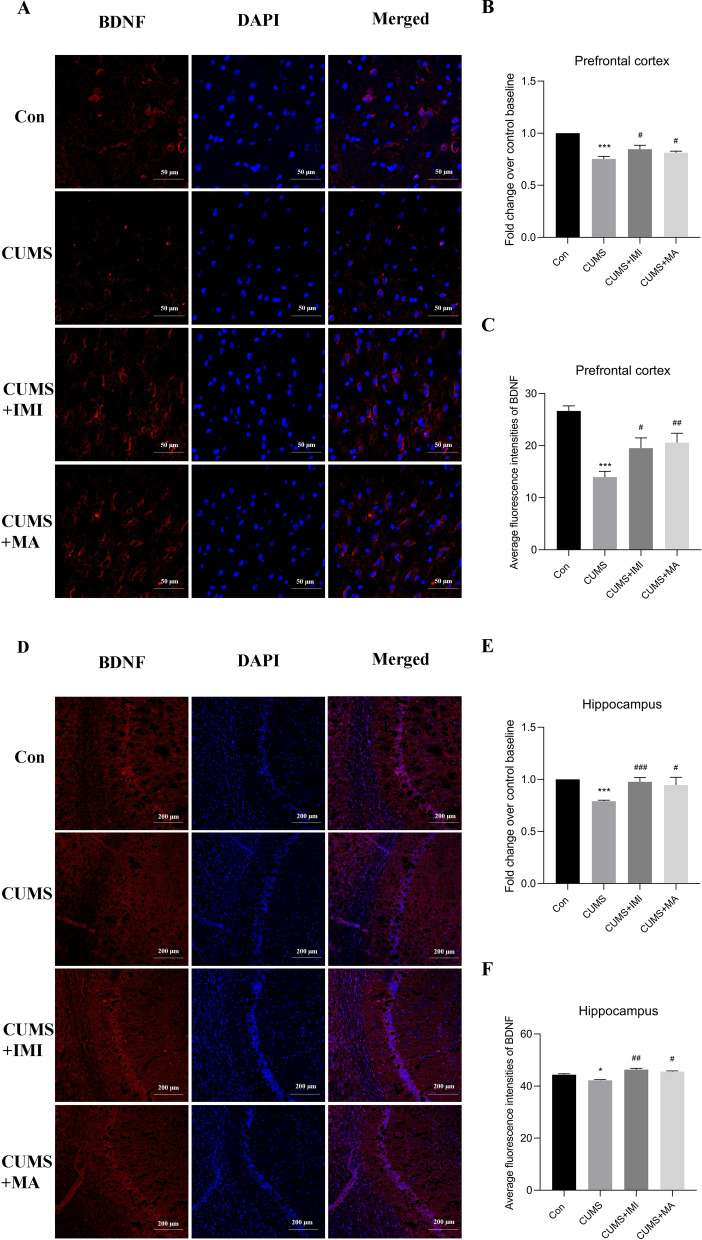
The relative levels of BDNF protein expression in different groups. **A** Immunofluorescence images of BDNF (red) and DAPI (blue) in the prefrontal cortex. The merged image is displayed in the right panel. Bar = 50 μm. **B** The relative levels of BDNF in prefrontal cortex in ELISA assay. **C** The average fluorescence intensity of BDNF in prefrontal cortex in **A**. **D** Immunofluorescence image of BDNF (red) and DAPI (blue) in the CA1 region of hippocampal. Bar = 200 μm. **E** The relative levels of BDNF in hippocampus in ELISA assay. **F** The average fluorescence intensity of BDNF in prefrontal cortex in FIGURE D. Data are presented as the mean ± SEM (n = 8). *p_*adj*_ < 0.05, ***p_*adj*_ < 0.001 versus the control group (Con); ^#^*p*_*adj*_ < 0.05, ^##^*p*_*adj*_ < 0.01, ^###^*p*_*adj*_ < 0.001 versus the CUMS group

Brain-derived neurotrophic factor (BDNF), a major member of the neurotrophin family, is one of the brain indicators of depression research. Depressive states, including elevated levels of proinflammatory cytokines in the brain, have been reported to be accompanied by down-regulation of BDNF expression in the brain [37, 38]. Therefore, BDNF levels in specific brain regions (prefrontal cortex and hippocampus) were examined. As illustrated in Fig. 5B, E, CUMS-induced group significantly reduced BDNF levels in prefrontal cortex and hippocampus in mice (*p*_*adj*_ = 3.97e−04 and *p*_*adj*_ = 5.79e−04). Meanwhile, the imipramine (*p*_*adj*_ = 0.03 and *p*_*adj*_ = 7.82e−04) and matrine (*p*_*adj*_ = 0.02 and *p*_*adj*_ = 0.04) treatment groups significantly increased BDNF levels in the prefrontal cortex and hippocampus compared with the CUMS group (ANOVA: Prefrontal cortex, F = 9.04, df = 3, *p* = 0.0002; Hippocampus, F = 18.53, df = 3, *p* = 7.36e−06).

Next, we further confirmed the expression level of BDNF in prefrontal cortex (Fig. 5A, C) and hippocampus (Fig. 5D, F) by immunofluorescence, which obtained consistent results. It was observed that the number of BDNF-positive cells and average fluorescence intensity in the prefrontal cortex (*p*_*adj*_ = 8.43e−04) and hippocampus (*p*_*adj*_ = 0.03) of mice exposed to CUMS for 6 weeks was significantly reduced compared to that of the control group, while CUMS mice treated with imipramine (*p*_*adj*_ = 0.02, *p*_*adj*_ = 0.007) or matrine (*p*_*adj*_ =0.003, *p*_*adj*_ = 0.02) showed a significant increase in the number of BDNF-positive cells and average fluorescence intensity (ANOVA: Prefrontal cortex, F = 8.97, df = 3, *p* = 0.0003; Hippocampus, F = 5.31, df = 3, *p* = 0.004). It indicates that matrine reduces the negative effect of CUMS on the relative content of BDNF, which is manifested in promoting the expression of BDNF protein in mice induced by CUMS.

### Correlation analysis between the related indicators of gut and brain in mice

**Fig. 6:**
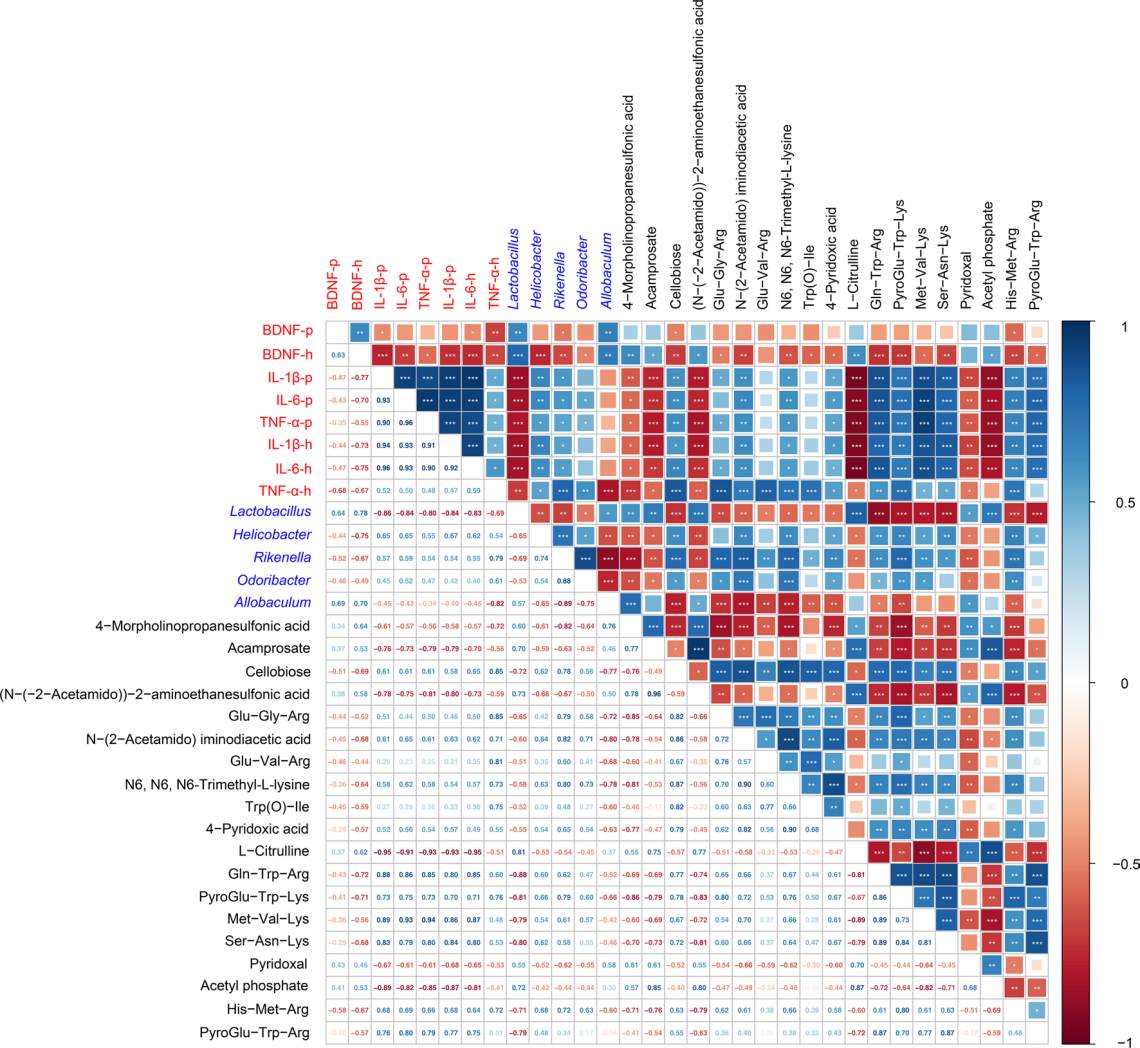
Spearman correlation between the related indicators of gut and brain. Axis label: red, BDNF and pro-inflammatory cytokines in the prefrontal cortex and hippocampus; blue, gut microbiota; black, gut metabolites. Numbers on the lower left area: value of correlation coefficient; symbols on the upper right area: results of correlation and significance test, *p < 0.05, **p < 0.01, ***p < 0.001

Spearman correlation analysis was carried out to evaluate the association among differential gut microbiota, differential gut metabolites, pro-inflammatory cytokines in specific brain regions (prefrontal cortex and hippocampus), and the expression levels of BDNF in specific brain regions. Gut microbiota was selected in the top 10 of the genus level and differences in LEfSe, and the differential metabolites were selected in the top 10 of Fig. 3C, E. As shown in Fig. 6, the level of BDNF in the prefrontal cortex and hippocampus displayed a positive correlation with the abundance of *Lactobacillus* and *Allobaculum*, and a negative correlation with the abundance of *Helicobacter*, *Rikenella*, and *Odoribacter*. The levels of pro-inflammatory cytokines (IL-1β, IL-6, and TNF-α) in the prefrontal cortex and hippocampus showed negatively correlated with the abundance of *Lactobacillus* and *Allobaculum*, and positively correlated with the abundance of *Helicobacter*, *Rikenella*, and *Odoribacter*. The level of BDNF in the prefrontal cortex and hippocampus was positively correlated with the content of Cellobiose, Glu-Gly-Arg, *N*-(2-acetamido) iminodiacetic acid, Glu-Val-Arg, N6, N6, N6-trimethyl-l-lysine, Trp(*O*)-Ile, 4-Pyridoxic acid, Gln-Trp-Arg, PyroGlu-Trp-Lys, Met-Val-Lys, Ser-Asn-Lys, His-Met-Arg, and PyroGlu-Trp-Arg, while negatively correlated with the content of 4-Morpholinopropanesulfonic acid Acamprosate, Acamprostate, (*N*-(-2-Acetamido))-2-aminoethanesulfonic acid, l-Citrulline, Pyridoxal, and Acetyl phosphate. What’s more, the relationship between the levels of pro-inflammatory cytokines (IL-1β, IL-6, and TNF-α) in the prefrontal cortex and hippocampus and the above-mentioned differential metabolites is just the opposite of the relationship between BDNF and the above-mentioned differential metabolites. Collectively, the correlation analysis results further provided the relationship between the related indicators of gut and brain, indicating that matrine improves CUMS-induced depression in mice via “gut–brain” communication.

## Disscussion

Previous evidence has shown that matrine possesses anti-cancer, anti-inflammatory, anti-oxidant, antiviral, antimicrobial, and neuro-protective properties. However, large doses of alkaloids have significant hepatotoxic and neurotoxic properties, which somewhat limit their clinical application. The dose of matrine selected in our study was within its safe dose range in vivo (10–160 mg/kg), which adequately avoided its toxicity [39, 40]. In the current study, to clarify the antidepressant effect of matrine, we established an animal model combining CUMS with separation to simulate human depression caused by long-term low-intensity stress [41], and successfully established a mouse depression model, as evidenced by four behavioral tests (SPT, FST, OFT, and NSFT) and the detection of monoamine neurotransmitters (5-HT, NE, and DA) in serum. Here, the four behavioral tests were used to evaluate the anhedonia, behavioral despair, exercise and exploration ability, and appetite in mice.

Studies have shown that long-term stress can lead to the imbalance of gut microbiota, whereas the classic antidepressant can alleviate the altered gut microbiota [42]. Depression-like behaviors in depression model mice exposed to stress are also related to changes in gut microbial composition [43, 44]. In our study, the structure of gut microbiota in the CUMS model group indeed changed significantly, manifested in the significant increase in the relative abundance of *Odoribacter*, *Rikenella*, *Streptococcus*, *Anaerotruncus*, *Clostridium*, and *Helicobacter*, however the trend was reversed after matrine treatment. It is known that the relative abundance of *Odoribacter* increases significantly after chronic and unpredictable mild stimulation [45]. *Odoribacter* in the intestine is significantly negatively correlated with brain BDNF and the levels of tight junction proteins in the brain and intestine [30, 31] and is significantly positively correlated with the levels of inflammatory cytokines in the brain and intestine, as well as the pro-inflammatory state of several metabolic and autoimmune diseases [30, 46, 47]. *Rikenella* is significantly negatively correlated with intestinal tight junction proteins (such as occludin and e-cadherin) and positively correlated with inflammatory cytokines, which is related to the intestinal mucosal barrier and the degree of inflammation [32–34]. Well, it was found that the relative abundance of *Streptococcus* and *Clostridium* in the intestine of major depressive disorder was significantly higher than normal person [48, 49]. *Streptococcus* belongs to the inflammation-promoting bacteria, and the increase in its relative abundance induces intestinal inflammation [50]. *Anaerotruncus* is positively correlated with anxiety and has been reported to increase in the feces of stressed animals, and increased in the feces of mice with intestinal barrier dysfunction [51, 52]. What’s more, it is reported that *Helicobacter* infection may cause glial to produce inflammatory products and affect the production of neurotrophic factors [53]. At the genus level, the abundance of *Helicobacter* in mice transplanted with fecal microbiota of depression-like mice was more significant than that in other groups [54]. Additionally, *Lactobacillus* is a differential microbiota in the control group (Fig. 2F). As we all know, genus *Lactobacillus* is one kind of probiotics in the intestinal, which is beneficial to alleviate intestinal inflammation and improve various neurological diseases [55–58]. In general, we observed that most of the significantly increased microbiota in the CUMS group have been confirmed to be positively related to inflammation and neurological diseases, especially depression and anxiety. However, matrine modulated and restored the composition of the gut microbiota associated with inflammation in depressed mice, suggesting that matrine mediates the regulation of intestinal microbes to improve depression-like behaviors.

As a systematic research method, metabolomics directly reflects the changes in the biochemical process and state of the body, plays a role in the identification and confirmation of pharmacological and disease models, and better captures the changes in endogenous metabolites caused by drugs [59]. Consistent with expectations, the CUMS procedure caused significant changes in most metabolites (Fig. 3C, E). From this, we further predict the metabolic pathways that it may participate in and affect. After analysis, we found that there are 4 metabolic pathways that are closely related to the treatment of depression, namely ABC transporters, biosynthetic of amino acids, neuroactive ligand-receptor interaction, and synaptic vesicle cycle. ABC transporters have been reported to be associated with depression. In fact, some ABC transporters are distributed throughout the brain and participate in the blood-brain barrier to regulate the inflow or outflow of various metabolites [60]. It is worth noting that in the shared metabolic pathways in Fig. 3D, F, the biosynthetic of amino acids has a higher impact. In addition, there are also changes in aminoacyl-tRNA biosynthesis, arginine biosynthesis, alanine, and aspartate and glutamate metabolism, which once again verify that the intestinal amino acid metabolism disorders are closely related to the pathophysiology of depression [61, 62]. In addition, the up-regulation of neuroactive ligand-receptor interaction contributes to the recovery of depression induced by CUMS. Notably, from the results, the up-regulation of the neuroactive ligand-receptor interaction pathway is beneficial to compensate for the low synaptic vesicle cycle, thereby promoting the release of neurotransmitters to improve CUMS-induced depression [63]. These reports coincide with our experimental results and also provide a theoretical basis for revealing the antidepressant mechanism of matrine. Therefore, we speculate that the CUMS procedure caused the dysfunction of amino acid metabolism in mice. While Matrine treatment alleviates the metabolic disorders and related symptoms caused by CUMS procedure *via* multiple metabolic pathways, thereby improving depression.

Anyway, our study confirmed that significant changes in a variety of amino acids and their metabolites are related to the intestinal barrier and inflammation. In view of the above results, we believe that it is necessary to further investigate the effect of matrine on indicators that reflect intestinal inflammation and permeability. Lipopolysaccharide (LPS) is a component of Gram-negative bacteria that can induce the production of IL-1β by binding to CD14 Toll-like receptor-4 (TLR4), and then IL-1β stimulates the production of other pro-inflammatory cytokines (such as TNF-α and IL-6), which in turn promotes the occurrence of intestinal inflammation and destroys the intestinal barrier, resulting in increased intestinal permeability [64]. The decrease in intestinal barrier function is accompanied by an increase in DAO, a marker of intestinal permeability [65]. Thus, the relative levels of DAO, LPS were tested. Additionally, pro-inflammatory cytokines can pass through the blood-brain barrier by second messengers or endothelial cells to carry out bidirectional communication between the central nervous system and the periphery [66]. Therefore, we next tested the relative levels of pro-inflammatory cytokines in prefrontal cortex and hippocampus, as well as in the serum of mice. It was found that matrine can restore intestinal permeability and intestinal barrier function, regulates the release of pro-inflammatory cytokines in the intestine and peripheral blood circulation, and relieves the inflammatory damage of the specific brain regions. This result is inseparable from the anti-inflammatory effect of matrine and its regulation of gut microbiota such as *Lactobacillus*.

Notably, in depression model mice, elevated levels of pro-inflammatory factors in the brain lead to impaired release of neurotrophic factors [67, 68]. Moreover, neuroinflammation promotes the down-regulation of BDNF expression in depressed patients and depression model animals [37, 38]. BDNF is one of the main members of the neurotrophic factor family, which is essential for neurogenesis and the pathogenesis of depression [69, 70]. In our research, the effect of matrine on BDNF is indeed opposite to that of pro-inflammatory factors, which suggests that the antidepressant mechanism of matrine involves ultimate upregulation of BDNF protein expression via regulating inflammatory factors. Furthermore, spearman correlation analysis corroborates the experimental results and further supports that matrine improves depression-like behaviors through “gut–brain” regulation. However, the causality of the bidirectional “gut–brain” communication has not been confirmed. In the next step, we will conduct a fecal microbiota transplantation experiment to verify the causal relationship of matrine to improve depression through “gut–brain” regulation, which is important to further explore its antidepressant mechanism. In addition, matrine is known to exhibit neuroprotective effects by inhibiting neuroinflammation and oxidative stress in various neurological disorders [71]. Moreover, it is able to regulate mitochondrial function and intercellular communication through multiple signaling pathways [72–76]. In the future, we will study in depth the direct targets and related molecular mechanisms of the antidepressant effects of matrine and further explore the possibility that oxidative stress and intercellular communication are involved in “gut–brain” communication to exert antidepressant effects.

## Conclusion

In conclusion, our research confirmed that matrine can regulate the composition of the gut microbiota and the disturbance of gut metabolites, restore intestinal permeability, reduce peripheral blood circulation and neuroinflammation, and increase the expression of BDNF protein, thereby effectively alleviating depression-like behaviors in CUMS-induced mice. This study provides a basis for exploring the mechanism of matrine to improve depression through the “microbiota–gut–brain” axis, and provides a new direction for matrine in the treatment of depression. Our future work will carry out fecal microbiota transplantation experiments to verify the causal relationship of matrine improving depression through “gut–brain” regulation.

## Supplementary Information

**Additional file 1: Figure S1.** The sucrose preference (%) of the control group, CUMS model group, imipramine group, and three different concentrations (-L: 15 mg/ml, -M: 30 mg/ml, and -H: 60 mg/ml) of matrine group at 0, 1, 4, and 6 weeks. Data are presented as the mean ± SEM (n = 8). *p_*adj*_ < 0.05, ***p_*adj*_ < 0.001 versus the control group (Con); ^#^*p*_*adj*_ < 0.05, ^##^*p*_*adj*_ < 0.01, ^###^*p*_*adj*_ < 0.001 versus the CUMS group. **Figure S2.** Comparison of alpha diversity of gut microbiota in mice with different treatment groups. **A** Chao1 index, **B** phylogenetic diversity, and **C** Shannon diversity. **Figure S3.** Boxplots showing differences in relative abundance of ASVs according to LEfSe analysis (select the top 50 ASVs at genus level). *p < 0.05 and **p < 0.01 versus the control group (Con); ^#^*p*_*adj*_< 0.05, ^##^*p*_*adj*_< 0.01, ^###^*p*_*adj*_< 0.001 versus the CUMS group. **Figure S4.** The heat map of 7 differential metabolites of amino acids (*p* < 0.05). **Figure S5.** Spearman correlation between neurotransmitters, depression-like behaviors, the differential gut microbiota, and the differential gut metabolites. Spearman’s rank correlation coefficient among 9 depression-related indicators, 5 gut microbiota, and 19 gut metabolites that differed significantly in abundance between different groups. Axis label: red, depression-related indicators; blue, gut microbiota; black, gut metabolites. Numbers on the lower left area: value of correlation coefficient; symbols on the upper right area: results of significance test, *p < 0.05, **p < 0.01, ***p < 0.001. **Table S1.**. The schedule of CUMS stressors. **Table S2.** Identification and change trend of differential metabolites in Fig. 3C, E.

## Data Availability

The datasets presented in this study can be found in online repositories. The names of the repository and accession number(s) can be found below: https://www.ncbi.nlm.nih.gov/, SRP329538.

## Abbreviations

CUMS: Chronic unpredictable mild stress
ELSA: Enzyme-linked immunosorbent assay
BDNF: Brain-derived neurotrophic factor
5-HT: Serotonin
NE: Norepinephrine
DA: Dopamine
DAO: Diamine oxidase
LPS: Lipopolysaccharide
IL-1β: Interleukin-1β
IL-6: Interleukin-6
TNF-α: Tumor necrosis factor-α
BW: Body weight
SPT: Sucrose preference test
FST: Forced swimming test
OFT: Open field test
NSFT: Novelty-suppressed feeding test
