# Supplementary material for: Matrine alleviates depressive-like behaviors via modulating microbiota–gut–brain axis in CUMS-induced mice
Source: J Transl Med. 2023 Feb 24;21:145. doi: 10.1186/s12967-023-03993-z (PMC9951532; doi:10.1186/s12967-023-03993-z)
Supplement: Supplementary file 1 — Additional file 1: Figure S1. The sucrose preference (%) of the control group, CUMS model group, imipramine group, and three different concentrations (-L: 15 mg/ml, -M: 30 mg/ml, and -H: 60 mg/ml) of matrine group at 0, 1, 4, and 6 weeks. Data are presented as the mean ± SEM (n = 8). *padj < 0.05, ***padj < 0.001 versus the control group (Con); #padj < 0.05, ##padj < 0.01, ###padj < 0.001 versus the CUMS group. Figure S2. Comparison of alpha diversity of gut microbiota in mice with different treatment groups. A Chao1 index, B phylogenetic diversity, and C Shannon diversity. Figure S3. Boxplots showing differences in relative abundance of ASVs according to LEfSe analysis (select the top 50 ASVs at genus level). *p < 0.05 and **p < 0.01 versus the control group (Con); #padj< 0.05, ##padj< 0.01, ###padj< 0.001 versus the CUMS group. Figure S4. The heat map of 7 differential metabolites of amino acids (p < 0.05). Figure S5. Spearman correlation between neurotransmitters, depression-like behaviors, the differential gut microbiota, and the differential gut metabolites. Spearman’s rank correlation coefficient among 9 depression-related indicators, 5 gut microbiota, and 19 gut metabolites that differed significantly in abundance between different groups. Axis label: red, depression-related indicators; blue, gut microbiota; black, gut metabolites. Numbers on the lower left area: value of correlation coefficient; symbols on the upper right area: results of significance test, *p < 0.05, **p < 0.01, ***p < 0.001. Table S1.. The schedule of CUMS stressors. Table S2. Identification and change trend of differential metabolites in Fig. 3C, E. [file 12967_2023_3993_MOESM1_ESM.docx]

***Additional file 1***

1. **Additional figures and tables**

**1.1 Additional figures**


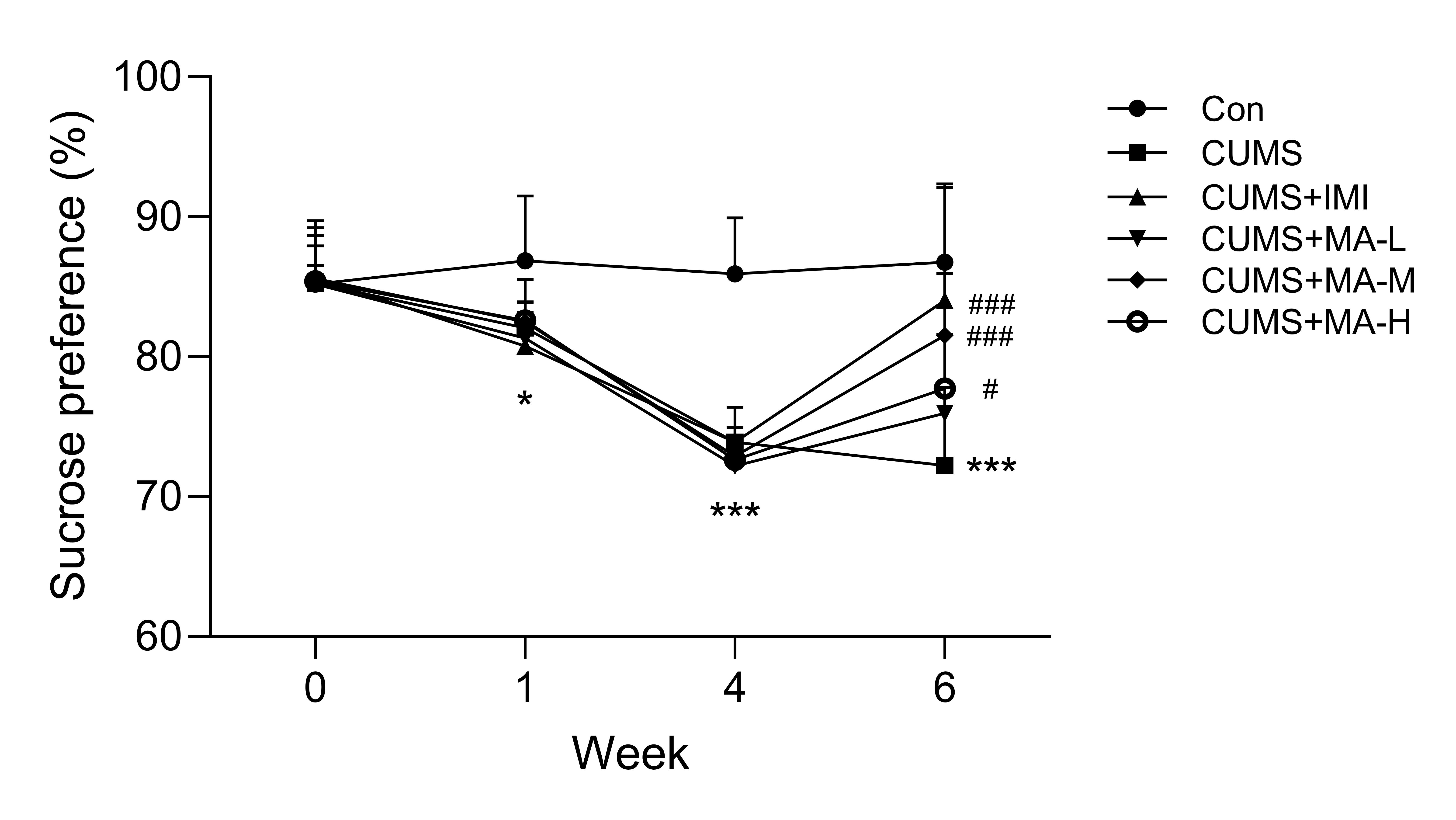


**Figure S1** The sucrose preference (%) of the control group, CUMS model group, imipramine group, and three different concentrations (-L:15 mg/ml, -M:30 mg/ml, and -H:60 mg/ml) of matrine group at 0, 1, 4, and 6 weeks. Data are presented as the mean ± SEM (n = 8). ^*^*p_adj_* < 0.05, ^***^*p_adj_* < 0.001 versus the control group (Con); ^#^*p_adj_* < 0.05, ^##^*p_adj_* < 0.01, ^###^*p_adj_* < 0.001 versus the CUMS group.


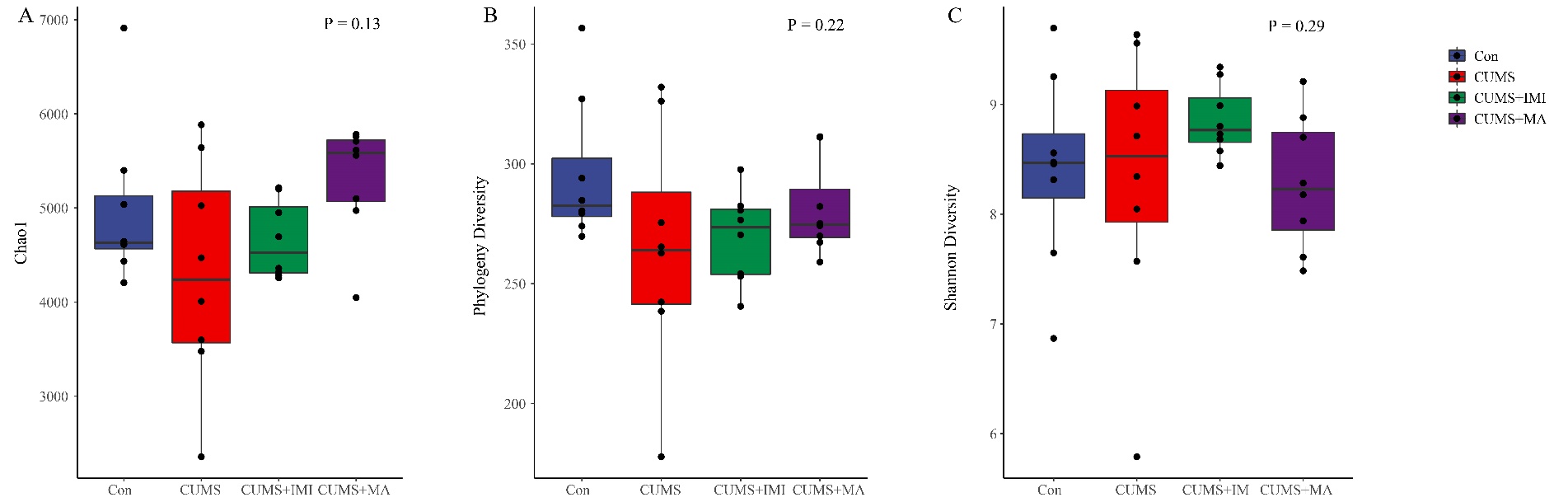


**Figure S2** Comparison of alpha diversity of gut microbiota in mice with different treatment groups. **(A)** Chao1 index, **(B)** Phylogenetic diversity, and **(C)** Shannon diversity.


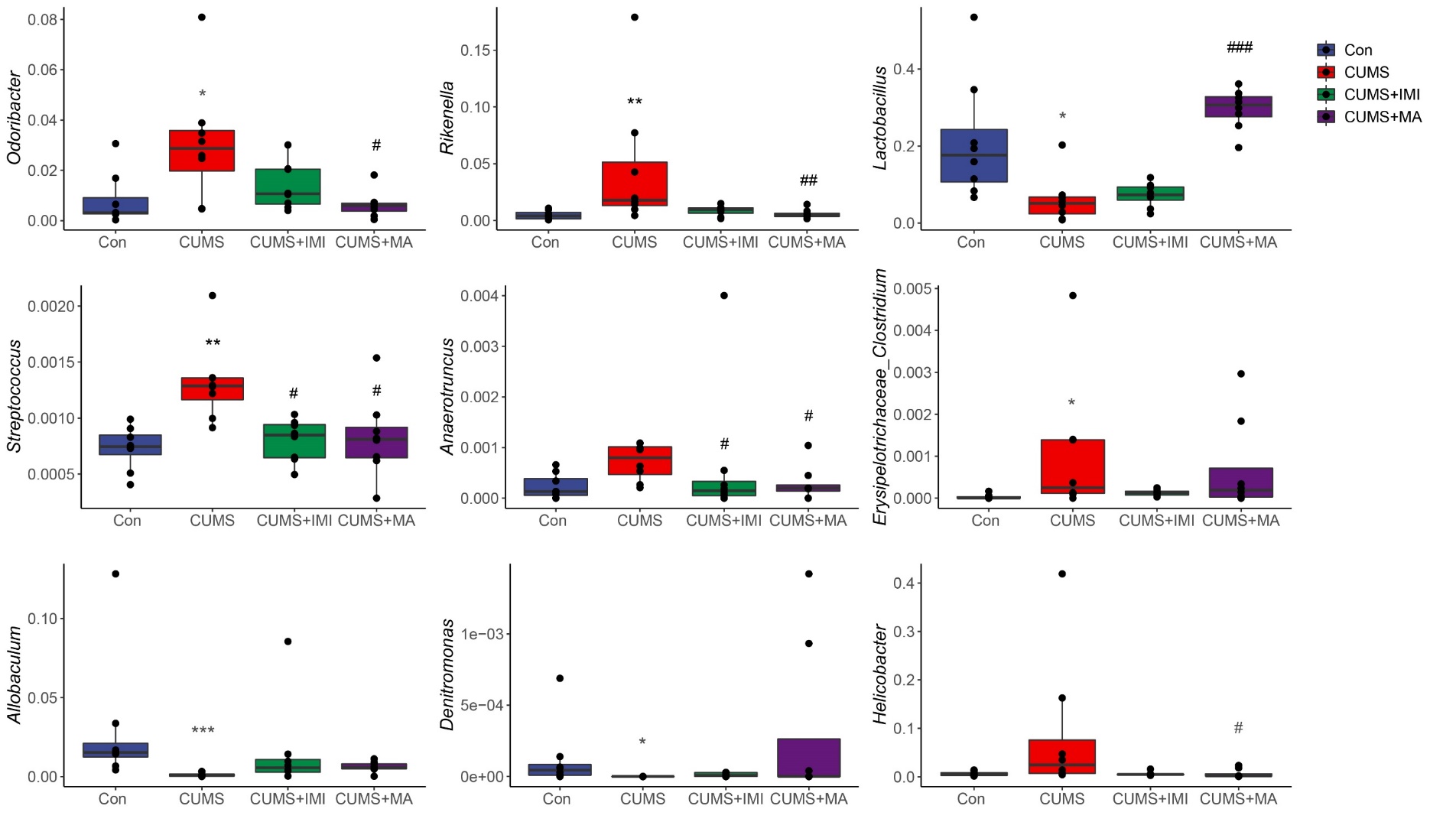


**Figure S3** Boxplots showing differences in relative abundance of ASVs according to LEfSe analysis (select the top 50 ASVs at genus level). ^*^*p* < 0.05 and ^**^*p* < 0.01 versus the control group (Con); ^#^*p_adj_* < 0.05, ^##^*p_adj_* < 0.01, ^###^*p_adj_* < 0.001 versus the CUMS group.


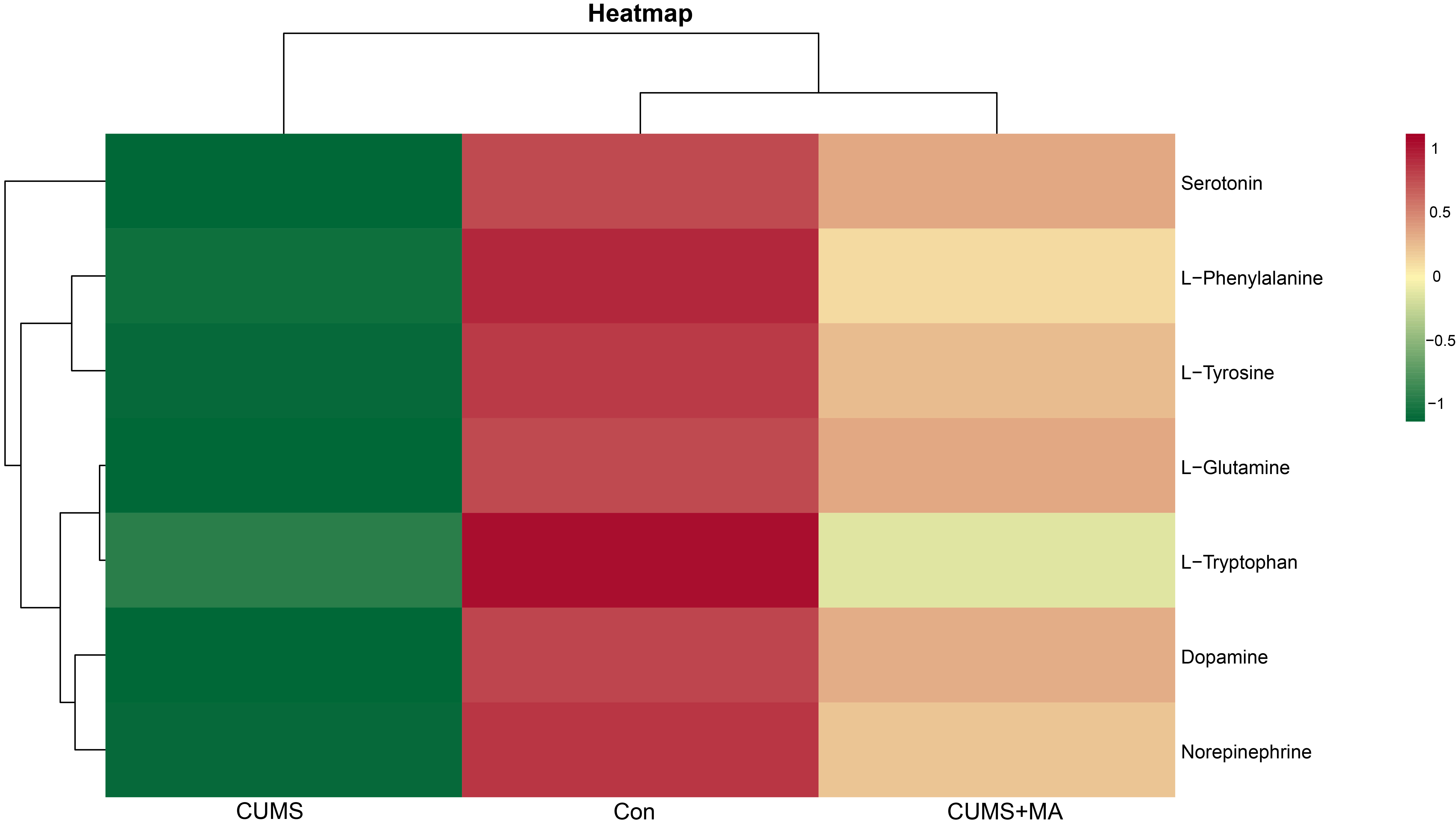


**Figure S4** The heat map of 7 differential metabolites of amino acids (*p* < 0.05)


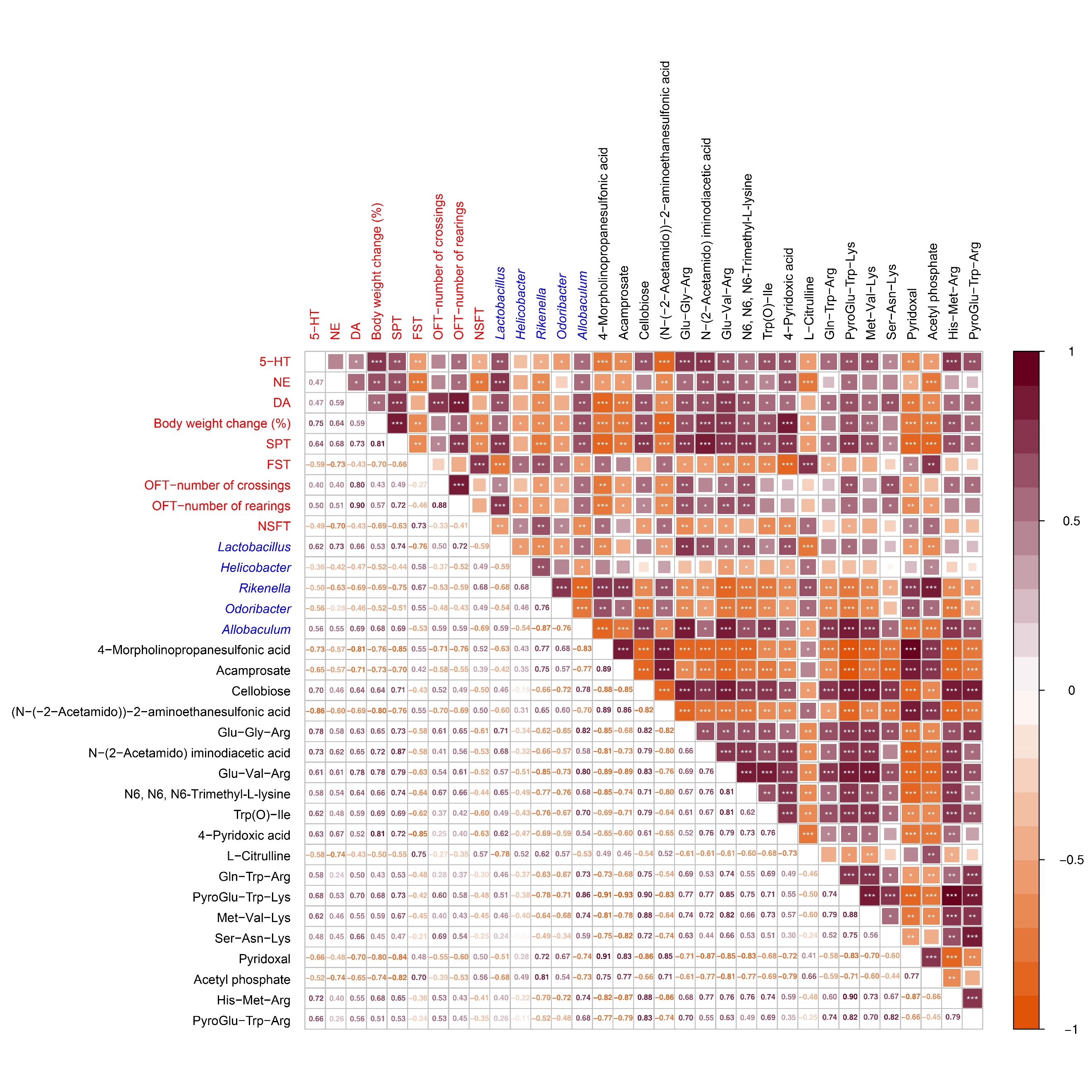


**Figure S5** Spearman correlation between neurotransmitters, depression-like behaviors, the differential gut microbiota, and the differential gut metabolites. Spearman’s rank correlation coefficient among 9 depression-related indicators, 5 gut microbiota, and 19 gut metabolites that differed significantly in abundance between different groups. Axis label: red, depression-related indicators; blue, gut microbiota; black, gut metabolites. Numbers on the lower left area: value of correlation coefficient; symbols on the upper right area: results of significance test, ^*^*p* < 0.05, ^**^*p* < 0.01, ^***^*p* < 0.001.

**1.2 Additional tables**

**Table S1** The schedule of CUMS stressors.

| Weeks | Days | | | | | | |
| --- | --- | --- | --- | --- | --- | --- | --- |
|  | Mon. | Tue. | Wed. | Thur. | Fri. | Sat. | Sun. |
| Week 1 | E+D | M | H+C | L+K | A+I | G+B | J+F |
| Week 2 | L+G | D+A | J+I | E+C | I+B | H+K | F+L |
| Week 3 | E+J | H+C | D+B | A+I | F+C | G+K | D+L |
| Week 4 | E+I | M | L+D | A+G | B+C | J+H | F+K |
| Week 5 | E+K | F+H | L+C | F+B | A+G | E+I | D+J |
| Week 6 | L+E | M | G+C | H+B | J+F | A+I | D+K |

A-food deprivation for 12 h; B-water deprivation for 12 h; C-lights on at night for 12 h; D-empty cage for 12 h; E-wet bedding for 12 h; F-confinement in a tube for 2 h; G-traffic noise (70-90 dB) for 2 h; H-cage tilting for 12 h (45°); I-exposure to a stroboscope for 12 h; J-foreign body stimulation for 2 h; K-crowding for 12 h (ten mice within one cage); L-level shaking for 15 min; M-food and water deprivation for 24 h.

**Table S2** Identification and change trend of differential metabolites in Figure 3C and E.

| No. | Metabolites | TR (min) | m/z | VIP | HMDB ID | CUMS/Con | CUMS+MA/ CUMS |
| --- | --- | --- | --- | --- | --- | --- | --- |
| 1 | 4-Morpholinopropanesulfonic acid | 90.63 | 208.06 | 1.83 | NA | ↑^***^ | ↓^***^ |
| 2 | Acamprosate | 82.93 | 108.03 | 1.83 | HMDB0014797 | ↑^***^ | ↓^***^ |
| 3 | Cellobiose | 451.78 | 360.15 | 1.81 | HMDB0000055 | ↓^***^ | ↑^***^ |
| 4 | (N-(-2-Acetamido))-2-aminoethanesulfonic acid | 158.23 | 181.03 | 1.81 | NA | ↑^***^ | ↓^***^ |
| 5 | Glu-Gly-Arg | 455.55 | 361.18 | 1.80 | NA | ↓^***^ | ↑^***^ |
| 6 | N-(2-Acetamido) iminodiacetic acid | 371.51 | 171.04 | 1.80 | NA | ↓^***^ | ↑^***^ |
| 7 | Glu-Val-Arg | 461.76 | 403.22 | 1.80 | NA | ↓^***^ | - |
| 8 | N6, N6, N6-Trimethyl-L-lysine | 144.00 | 187.13 | 1.79 | HMDB0001325 | ↓^***^ | ↑^***^ |
| 9 | Trp(O)-Ile | 391.99 | 316.15 | 1.79 | NA | ↓^***^ | - |
| 10 | 4-Pyridoxic acid | 134.06 | 182.04 | 1.79 | HMDB0000017 | ↓^***^ | ↑^**^ |
| 11 | UDP-D-Galactose | 482.15 | 584.08 | 1.79 | HMDB0000302 | ↑^***^ | - |
| 12 | Rosiglitazone | 258.96 | 358.11 | 1.78 | HMDB0005031 | ↓^***^ | ↑^***^ |
| 13 | D-Pipecolinic acid | 508.01 | 281.15 | 1.77 | HMDB0005960 | ↓^***^ | ↑^**^ |
| 14 | Galactinol | 448.10 | 683.21 | 1.77 | HMDB0005826 | ↓^***^ | ↑^**^ |
| 15 | D-glucosamine 6-phosphate | 45.96 | 259.06 | 1.77 | HMDB0001254 | ↓^***^ | ↑^*^ |
| 16 | N-Acetylserotonin | 61.29 | 219.11 | 1.76 | HMDB0001238 | ↓^***^ | ↑^***^ |
| 17 | D-Maltose | 428.07 | 341.10 | 1.76 | HMDB0000163 | ↓^***^ | ↑^***^ |
| 18 | PyroGlu-Trp-Lys | 458.05 | 444.21 | 1.75 | NA | ↓^***^ | ↑^***^ |
| 19 | His-Met-Arg | 314.94 | 443.23 | 1.75 | NA | ↓^***^ | ↑^***^ |
| 20 | N4-Acetylcytidine | 448.82 | 327.11 | 1.74 | HMDB0005923 | ↓^***^ | ↑^***^ |
| 21 | L-Citrulline | 434.99 | 176.09 | 1.83 | HMDB0000904 | ↑^***^ | ↓^***^ |
| 22 | Gln-Trp-Arg | 459.32 | 489.26 | 1.82 | NA | ↓^*^ | ↑^***^ |
| 23 | Met-Val-Lys | 368.16 | 377.21 | 1.81 | NA | ↓^***^ | ↑^***^ |
| 24 | Ser-Asn-Lys | 513.75 | 348.17 | 1.80 | NA | ↓^**^ | ↑^***^ |
| 25 | Pyridoxal | 120.03 | 168.06 | 1.80 | HMDB0001545 | ↑^**^ | ↓^***^ |
| 26 | Acetyl phosphate | 471.36 | 278.97 | 1.79 | HMDB0001494 | ↑^**^ | ↓^***^ |
| 27 | PyroGlu-Trp-Arg | 486.33 | 472.22 | 1.79 | NA | - | ↑^***^ |
| 28 | Asn-Glu-Lys | 460.89 | 390.18 | 1.79 | NA | ↓^**^ | ↑^***^ |
| 29 | Tyr-Tyr-Arg | 465.48 | 501.25 | 1.78 | NA | ↓^***^ | ↑^***^ |
| 30 | Thr-Gly-Lys | 523.33 | 305.16 | 1.78 | NA | - | ↑^***^ |
| 31 | Histamine | 461.86 | 112.08 | 1.78 | HMDB0000870 | ↑^***^ | ↓^***^ |
| 32 | Thr-Ser-Lys | 511.67 | 376.20 | 1.78 | NA | ↓^**^ | ↑^***^ |
| 33 | Dacarbazine | 291.75 | 224.13 | 1.77 | HMDB0014989 | ↓^**^ | ↑^***^ |
| 34 | Phe-Phe | 189.05 | 313.15 | 1.77 | HMDB0013302 | - | ↑^***^ |
| 35 | Tyr-Ser-Lys | 496.25 | 396.19 | 1.77 | NA | ↓^***^ | ↑^***^ |
| 36 | Ser-Thr-Lys | 488.76 | 335.19 | 1.76 | NA | - | ↑^***^ |
| 37 | Tyr-Thr-Lys | 507.57 | 410.21 | 1.74 | NA | ↓^***^ | ↑^***^ |

Note: TR, retention time; VIP, variable importance in projection, this value was obtained from the OPLS-DA model with a threshold of 1. Changes in differential metabolites are marked as (↓) down-regulation and (↑) up-regulation. ^*^*p* < 0.05, ^**^*p* < 0.01, and ^***^*p* < 0.001.
